# Isolation and Biological Properties of the Natural Flavonoids Pectolinarin and Pectolinarigenin—A Review

**DOI:** 10.3390/antibiotics9070417

**Published:** 2020-07-16

**Authors:** Thamere Cheriet, Balkeis Ben-Bachir, Oumelkhir Thamri, Ramdane Seghiri, Ines Mancini

**Affiliations:** 1 Unité de Valorisation des Ressources Naturelles, Molécules Bioactives et Analyse Physicochimiques et Biologiques (VARENBIOMOL), Université des Frères Mentouri, 25000 Constantine, Algeria; seghiri25000@yahoo.fr; 2 Département de Chimie, Faculté des Sciences, Université Mohamed Boudiaf-M’sila, 28000 M’sila, Algeria; bebabalkisbeba@gmail.com (B.B.-B.); murielvapula1@gmail.com (O.T.); 3 Laboratorio di Chimica Bioorganica, Dipartimento di Fisica, Universita’ di Trento, I-38123 Povo-Trento, Italy

**Keywords:** biological activities, antitumor, antidiabetic, anti-inflammatory

## Abstract

Flavonoids are metabolites widely distributed in plants and commonly present in foods, such as fruits and vegetables. Pectolinarin, which belongs to the flavone subclass, has attracted considerable attention due to its presence in many medicinal plants. It has turned out to be a good biological agent especially due to its antioxidant, anti-inflammatory, antidiabetic, and antitumor activities, evaluated both in vitro and in vivo. Its aglycone, the metabolite pectolinarigenin, is also known for a series of biological properties including anti-inflammatory and antidiabetic effects. In the first overview on the two metabolites here presented, their collection, isolation and the results of their biological evaluation are reported.

## 1. Introduction

Nature is recognized as a source of molecules with relevant potential pharmaceutical applications, also in the 21st century. Among the various phytochemicals, flavonoids have attracted and are still attracting most of the attention due to their notable biological benefits. Based on several evidences, flavonoids have been associated with the role of preventing and managing current diseases such as cancers, diabetes, and cardiovascular disorders. More than 13,000 flavonoids were isolated and identified from plants, some of which like quercetin, kampferol and scutellarin showed potent pharmacological effects [1] and, therefore, are promising for new drugs.

**Figure 1:**
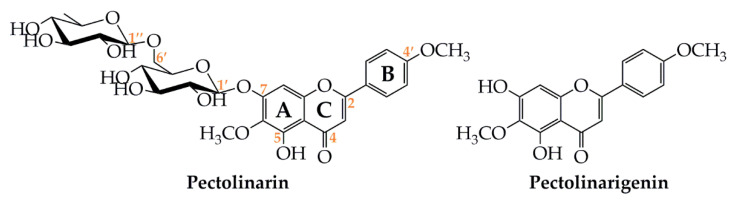
Molecular structures of pectolinarin and pectolinarigenin.

The glycosylated flavone pectolinarin was first isolated from *Linaria vulgaris* [2], a known medicinal Chinese herb used for the internal treatement of digestion problems and urinary disorders, in the external treatment of haemorrhoids, venous skin ulcer, as well as for the washing of festering wounds and skin rashes. It has also displayed anti-inflammatory effect [3] and has been used to treat coughs and asthma [4]. The structure of pectolinarin was determined to be a rutinoside conjugate of pectolinarigenin (=5,7-dihydroxy-4,6-dimethoxyflavone, C_17_H_14_O_6_) at the 7-*O* position (pectolinarigenin-7-*O*-rutinoside, C_29_H_34_O_15_) (Figure 1).

Later, pectolinarin and its aglycone pectolinarigenin were identified as the major constituents in many medicinal herbs from different genera around the world. Several studies reported so far prove that the presence of these two flavones has an important role in affecting the biological properties of the following herbs: i) the Korean herb *Cirsium setidens* (Dunn) Nakai employed for the treatment of hemostasis, hematemesis, hematuria and hypertension [5]; ii) the Chinese herb *Cirsium chanroenicum* used for detoxification, to treat fever and to enhance blood circulation [6]; iii) *Cirsium japonicum* DC. employed as an anti-hemorrhagic and uretic agent, as well as prescribed to treat liver and uterine tumours, and leukemia [7]; iv) *Kickxia ramosissima* (Wall.) Janch., used in Pakistan folk medicine as diuretic and against kidney stones [8], fever and rheumatism [9], and during management of snake and scorpion bites [10]; v) *Lantana camara* L., used for the treatment of various human ailments, such as ulcers, malaria, influenza, tumors, swellings, bilious fever, eczema eruptions, stomach ache, toothache, and as antiseptic for wounds [11]; and vi) *Picnomon acama* (L.) Cass., used in Greek folk medicine as hemostatic and spasmolytic agent [12].

Due to the structural similarity of pectolinarigenin to known potent flavonoids such as acacetin (5,7-dihydroxy-4′-methoxyflavone, C_16_H_12_O_5_), hispidulin (4′,5,7-trihydroxy-6-methoxyflavone, C_16_H_12_O_6_) and scutellarein (5,6,7,4′-tetrahydroxyflavone, C_15_H_10_O_6_) and based on the numerous data reported for both pectolinarin and pectolinarigenin, the aim of this work is to provide a comprehensive overview focusing on their isolation, pharmacological aspects and therapeutic potential. 

## 2. Isolation of Pectolinarin and Pectolinarigenin

Over the last 113 years from its first report [2], pectolinarin was isolated in most cases from the aerial parts of 87 plants belonging to 29 differents genera distributing widely around the world. Most of these plants are used in folk medicine in different parts of the world. Table 1 gathers the 87 plants from which pectolinarin was isolated.

**Figure 2:**
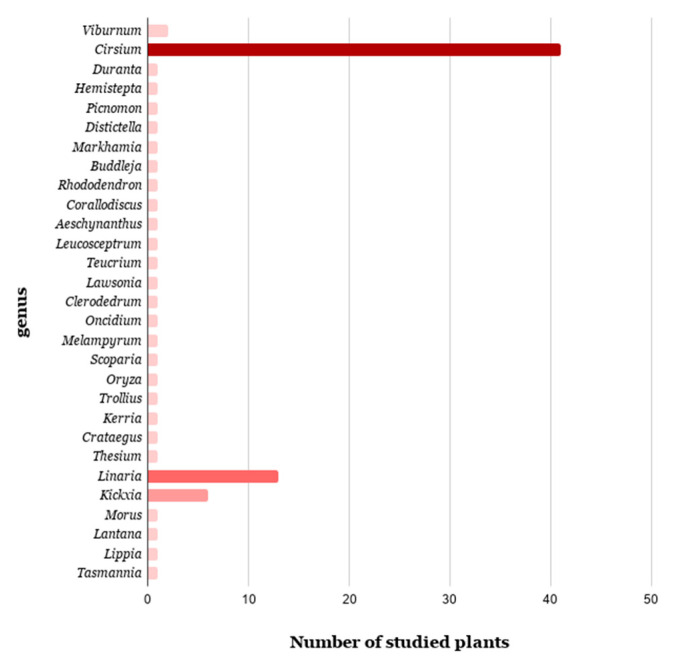
Number of plants investigated for the presence of pectolinarin, classified according to their genus.

**Figure 3:**
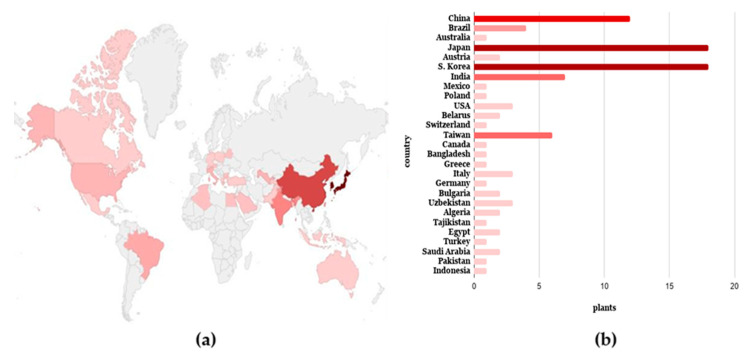
Worldwide distribution of the plants containing pectolinarin (**a**), with indication of their number from each country (**b**).

*Cirsium* emerges as the most known genus for the presence of pectolinarin (47.13%), including 41 plant species (Figure 2). The relevant amount of this metabolite in many of these medicinal plants was detected and quantificated by HPLC-UV analysis on methanolic extracts of *Cirsium* plants [43], including *C. japonicum* (1.04 ± 0.01 mg/g), known in the Chinese medicine to treat liver and uterine cancers, and leukemia [7]; *C. setidens* (33.9 ± 0.5 mg/g), which grows only in Korea and is employed to treat hemorrhage, hypertension and diabetes [23,95]; *C. chlorolepis* (110.7 ± 13.4 mg/g), whose roots are used in Southwest China as a remedy in the cure of fractures and haematuria [96]; *C. nipponcium* (61.5 ± 0.6 mg/g), as diaphoretic and vulnerary in Chinese herbal medicine [97], and *C. chanroenicum* (49.4 ± 7.1 mg/g), also reported in Chinese medicine for detoxification, to treat fever and to enhance blood circulation [6]. Figure 3 presents the distribution of the plants studied evaluated for the presence of pectolinarin, pointing out their countries of origin. 

The commonly applied methods to isolate secondary metabolites from plants include maceration, polar solvent extraction and liquid-liquid exctration using solvents at different polarity to obtain selected separations. In particular, due to their polar property, flavonoids, and especially the polyhydroxylated or glycosides ones, require the use of polar solvents. 

The following reports have been taken as some case studies on the isolation and purification of pectolinarin. With the aim to isolate flavonoids and mainly this metabolite in high purity from *C. setidens*, Cho et al., [98], described a method which started from drying and slicing the plant material, followed by extraction with a hydroethanolic solution (40 to 90%). The solution obtained was concentrated and then dissolved in methanol. The extract was separated into supernatant and precipitate by centrifugation, then the supernatant was crystallized, washed with methanol and dried to obtain 85–90% the pure metabolite. Pectolinarin was quantitatively detected together with other constituents in nine Korean native composite herbs. It showed the highest amount in hydromethanolic extracts from *Cirsium setidens* and *C. pendulum*, with values of 73.3 and 76.7 mg/g respectively [24]. In detail, it was eluted at a retention time of 8.2 min by the HPLC analysis (C18 column) using water/acetonitrile with a gradient system starting from 75:25, till pure acetonitrile [43]. Lastly, it was detected at retention time 25.7 min by a reversed C18 chromatographic analysis eluting with water, methanol and acetonitrile, with 0.05% (*v/v*) of trifluoroacetic acid (TFA) also added in each solvent, with gradient elution at a flow rate of 1 mL/min [24].

Pectolinarigenin is the aglycone part of pectolinarin, which is obtained by hydrolysis reaction [73]. It is also a natural product, isolated and identified from 136 plantes of 71 differents genera. The data are summarized in Table 2, indicating that pectolinarigenin was isolated from 20 different families, especially from Asteraceae with 33 genera and 64 species (47.1%), Lamiaceae with 9 genera and 19 species (14%) and Verbenaceae with 4 genera and 10 species (8%).

**Figure 4:**
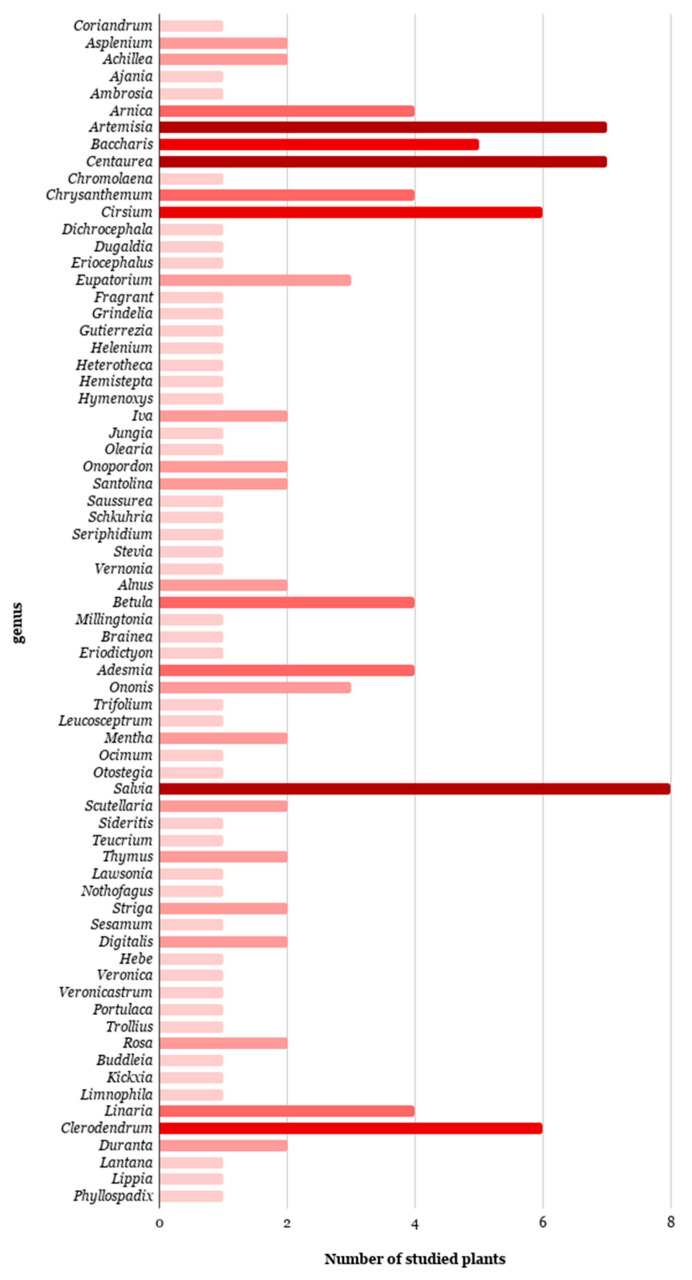
Number of plants investigated for the presence of pectolinarigenin, classified according to their genus.

Pectolinarigenin proved to be more distributed in the vegetable kingdom than pectolinarin, and this evidence can be explained assuming that not all plants have the *O*-glycosylation enzymes. This is evident comparing the distributions of pectolinarin in Figure 2 and of pectolinarigenin reported in Figure 4. According to the APG III classification [221], most families from where pectolinarin and pectolinarigenin were isolated belong to the groups Asterids (Adoxaceae, Asteraceae, Bignoniaceae, Boraginaceae, Ericaceae, Gesneriaceae, Lamiaceae, Orobanchaceae, Plantaginaceae, Scrophulariaceae and Verbenaceae), Rosids (Betulaceae, Fabaceae, Lythraceae, Moraceae, Nothofagaceae and Rosaceae), Magnoliids (Winteraceae), and Core eudicots (Santalaceae). This may suggest that these genera have the same enzyme responsible for the biosynthesis of these two flavones, although the biosynthesis pathway itself has not been established yet.

In most works, pectolinarigenin was isolated from the non-polar fractions of the plant extracts, mainly working on the aerial parts. Some reports indicate the possibility of analysing this metabolite by HPLC technique under detailed conditions. An analytical HPLC study deals with the identification of 13 active compounds in *Hemistepta lyrata* methanolic extract, where pectolinarigenin was eluted at 39 min on a reversed phase C18 columun and gradient elution using water and a mixture of methanol and acetonitrile, acidified by adding 0.05% (*v/v*) of trifluoroacetic acid (TFA) [45]. Jeong et al. reported the quantitative analysis of pectolinarin and pectolinarigenin as markers of *Cirsium setidens*, with elution of pectolinarigenin at 76 min from a C18 column using a 0.5 mL/min flow of water/methanol containing 0.5% phosphoric acid [222].

## 3. Biological Activities of Pectolinarin and Pectolinarigenin

**Figure 5:**
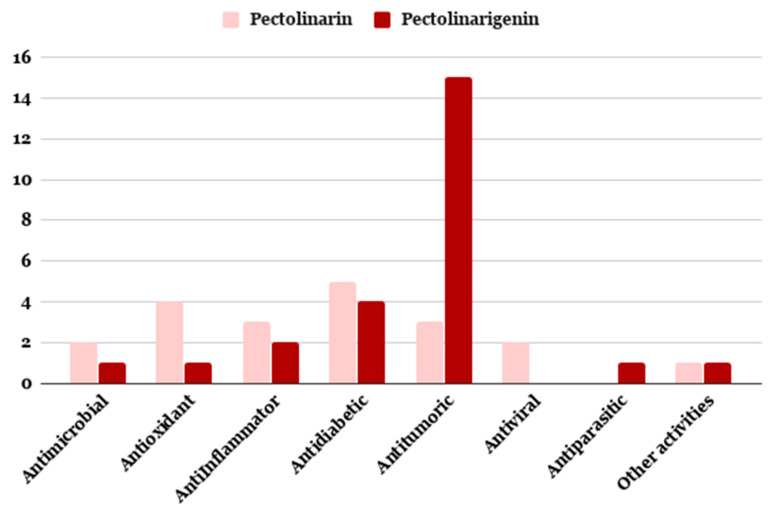
Distribution of the biological activities reported for pectolinarin and pectolinarigenin.

Most studies on pectolinarin and pectolinagenin herein overviewed are devoted to their isolation, purification and structural elucidation, with their biological evaluation reported in a series of works summarized in Figure 5, according to their kind of actvivities. The beneficial effects of flavones have been attributed mainly to their antioxidant capacity, including direct free radical scavenger and metal-chelating properties. Additionally, they have shown broader indirect antioxidant effects through their ability to interact and modulate antioxidant enzyme activities [223]. It occurs also for the two metabolites of our interest.

### 3.1. Antimicrobial Activity

Poultices, infusions, balms, and spices containing flavonoids as antibacterial active constituents have been used in many cultures for centuries [224]. Traditional uses include the treatment and prevention of various infectious and toxin-mediated diseases, e.g., sores and wound infections [225], acne, respiratory infections [226], gastrointestinal disease [227], and urinary tract infections [228]. Therefore, it is not surprising that this family of metabolites has attracted extensive research as new antimicrobials in our time. This is due to the recent global problem of antibiotic resistance, which focuses the attention on an urgent need to replenish our drug arsenal with new more potent agents [229].

It was observed that flavonoids’ antibacterial activity can be exerted in three ways: directly killing the bacteria, by activating synergistically with antibiotics and attenuating the bacterial pathogenicity [224]. It is worth mentioning that the flavonoids have shown inhibitory activity against the efflux pump of methicillin-resistant *Staphylococcus aureus* [230], and restrained the synthesis of peptidoglycan and ribosome in the cells of amoxicillin-resistant *Escherichia coli* [231]. They have also inhibited different kinds of *β*-lactamases produced by bacteria, which are the key enzymes acting to disable the common antibiotics [232,233]. A study on the methanolic extract (containing five flavonoids from which they were then isolated) obtained from *Lawsonia inermis*, showed that pectolinarin and pectolinarigenin were active against three bacterial (*Escherichia coli*, *Staphylococcus aureus*, *Klebsella pneumonia*) and three fungi (*Aspergillus niger*, *Aspergillus flavus*, *Candida albicans*) microorganisms. By a mean inhibition zone test on the tested metabolites, pectolinarin displayed the most remarkable inhibition agaist all tested strains, especially *S. aureus* (18 and 24 mm at 50 and 100 μg, respectively) and *E. coli* (22 and 27 mm at 50 and 100 μg, respectively). These data suggest that pectolinarin can be considered an interesting antimicrobial agent [55]. A study on 100 plant-derived natural products against *Mycobacterium tuberculosis* (Mtb) proteasome revealed a strong inhibition by pectolinarin (88.7%), with an IC_50_ value of 49.96 μM at the 200 μM dose. This resulted as the second highest value after baicalein (45.7 μM), in comparison with the well-known synthetic proteasome inhibitor peptide aldehyde MG132 (28 μM), suggesting that pectolinarin has a potent activity against Mtb [234]. Several structure-activity relationship studies on flavones as antibacterial agents indicate that: (i) a 5,7-dihydroxyl substitution is a positive indicator of the activity, (ii) an additional hydroxyl group at 4’ position greatly increases the effect, while (iii) the methylation of these hydroxyl groups reduces the activity to different degrees [235,236,237]. Regarding these data, we can suggest that the activity observed for pectolinarin is related to the presence of 5,7-dihydroxyl groups; however, the role of sugar moiety was not defined.

### 3.2. Antioxidant, Anti-Inflammtory and Antidiabetic Activities

Flavonoids are potent antioxidants, whose activity is by delaying, preventing or removing oxidative harm to a target molecule [238]. Their comprehensive action mode includes: (i) free radical quenching, (ii) metal ion chelation, (iii) suppressing the enzymes associated with free radical generation, and (iv) stimulation of internal antioxidant enzymes [239].

Some reports deal with the antioxidant properties of pectolinarin or pectolinarigenin based on the biological data on the plants extract from where they were isolated. The in vitro antioxidant activity of some *Cirsium* methanolic extracts (*C. setidens*, *C. lineare*, *C. nipponicum*, *C. pendulum*, *C. japonicum* and *C. chanroenicum*) containing pectolinarin as major metabolite, was evaluated using the 2,2-diphenyl-1-picryl-hydrazyl-hydrate (DPPH) free radical method. In comparison with ascorbic acid (2.7 ± 2.6 μg/mL), *C. setidens* and *C. chanroenicum* extracts exhibited the most remarkable effect, with IC_50_ values of 4.6 ± 1.8 and 5.9 ± 1.7 μg/mL, respectively. The data from ONOO^−^ scavenging test on *C. chanroenicum*, *C. lineare* and *C. setidens* methanolic extracts, displayed interesting results, with IC_50_ values of 1.3 ± 1.3, 1.9 ± 0.1 and 2.1 ± 2.4 μg/mL, respectively. In detail, *C. chanroenicum* extract displayed a stronger effect than penicillamine (IC_50_ 1.3 ± 0.1 μg/mL). These results suggest that flavonoids, especially pectolinarin, are the molecules responsible for the observed effects [19]. A column chromatography purification was performed on the extract. Based on the promising antioxidant and hepatoprotective activities observed for the *n*-butanol fractions from the methanolic extract of *Cirsium setidens* leaves [240], a column chromatography purification was performed on the extract. Pectolinarin was identified as the major metabolite and further hydrolyzed to yield pectolinarigenin. The hepatoprotective efficacy of the two pure flavones was evaluated in a rat model of hepatic injury caused by D-galactosamine (GalN). A 2-week administration (10, 20 mg/kg, p.o.) of both metabolites significantly decreased the activity levels of serum aspartate transaminase (AST), alanine transaminase (ALT), alkaline phosphatase (ALP), and lactate dehydrogenase (LDH), indicating that they had hepatoprotective activity. Both pectolinarin and pectolinarigenin increased also the activity levels of the tripeptide glutathione and of the enzymes glutathione reductase, γ-glutamylcysteine synthetas, glutathione S-transferase, and superoxide dismutase. A significant effect was only seen in superoxide dismutase (SOD) action (7.6 ± 0.4 and 6.9 ±0.3 μmol/mg protein, at the dose 10 mg/kg, respectively). It was concluded that both pectolinarin and pectolinarigenin exhibit hepatoprotective activity, mainly via SOD antioxidant mechanism [23]. Notably, these results were comparable to the ones reported for hispidulin which is structurally very similar to pectolinarigenin (with an OH replacing the OMe groupin C-4′, Figure 1), where the potent hepatoprotective activity shown by hispidulin using varied doses (50–300 mg/kg) [241,242,243] must be compared with the significant activity of pectolinarigenin at a lower dose (10 and 20 mg/kg). However, further studies are necessary to determine the action mechanism of pectolinarin and pectolinarigenin.

A study of *Cirsium setidens* aqueous extract and its major metabolite pectolinarin (2.8 ± 0.1 mg/g of plant extract), showed that both inhibited the accumulation of lipids during the adipogenesis of 3T3-L1 cells by a negative regulator of the factor adipogenic transcription. Supplement for *C. setidens* extract suppressed body weight in C57BL/6 mice fed a high-fat diet and reduced plasma levels of total cholesterol, triglycerides, insulin, and glucose. The results indicated that the extract enriched with pectolinarin can be considered a good source of natural antioxidants and anti-obesity ingredients [244]. In the light of the data on *Lantana camara* leaves extract, which displayed a strong inhibition on DPPH radicals and antioxidant effects (IC_50_ 7.6 μg/mL), its chemical investigation led to identify pectolinarin in a high amount [90], suggesting that just this metabolite could be responsible for the remarkable antioxidant activity of the plant extract.

Bhakta and Ganjewala [245] examined the relation between leaves position (from apex to base) and the corresponding levels of phenolics, flavonoids and proanthocyanidins. It was observed that premature leaves of *L. camara* are very active in the biosynthesis and accumulation of secondary metabolites and, hence, exhibit antioxidant activity with a 62% of DPPH scavenging activity. This activity was ascribed to the high presence of phenolics (0.038 μg/mL), proanthocyanidins (181.6 mg/mL) and flavonoids (21.7 μg/mL). Another research on the neuroprotective effects of pectolinarin proved its capacity to reduce oxidative stress-induced cell death and intracellular reactive oxygen species (ROS) formation in human neuroblastoma SK-N-SH cells [246]. This evidence can be significant because oxidative stress is one of the main factors implicated in the progression of neurodegenerative diseases such as Alzheimer’s, Parkinson’s and ischemic stroke [247,248]. Recently, Wu and Liang [138] examined the role of pectolinarigenin in spinal cord injury (SCI), a devastating neurological injury that frequently leads to neurological defects and disabilities. Pectolinarigenin significantly improved functional recovery, and reduced tissue loss and neuronal apoptosis, suggesting that this molecule may exert a protective effect against SCI in rats, potentially by a potential inhibition of neuronal apoptosis. These observations are in line with neurological data obtained for flavones that are structurally similar to pectolinarigenin. Nam et al. [249] reported that jaceosidin (with OMe group at C-3′ and OH on C-4′) ameliorates neuroinflammation in a mouse model of experimental allergic encephalomyelitis, whereas eupatilin (with two OMe groups at C-3′ and C-4′) showed neuroprotective activity against transient global cerebral ischemia in mice by increased Akt (Protein Kinase B) phosphorylation [250].

It is reported that the imbalance between oxidants and antioxidants is responsible for the damage of lipids, proteins and nucleic acid, causing several human diseases, like neurodegenerative disorder, lung and cardiovascular diseases, diabetes, and atherosclerosis [251]. Furthermore, an overproduction of ROS attack tissues can cause an inflammatory response that can become chronic [252]. Inflammation involves the development of a series of phenomena, which includes edema, pain, erythema, and an increase in temperature/fever. The development of inflammation is initiated by the release of different mediators through many cellular sources. They include the mediators produced by cells (i.e., serotonin, histamine, cytokines and chemokines, nitric oxide, ROS) and lipid mediators (such as prostanoids, leukotrienes and lipoxins), in addition to those derived from components present in the plasma (bradykinin) and members of the coagulation system. Flavonoids interfere with these molecules at various degrees, depending on the concentration/doses of flavonoids used in vitro and in vivo [253].

A study was carried out comparing the analgesic and anti-inflammatory effects in mice and rats of pectolinarin and linarin, isolated from the aqueous extracts of *Cirsium subcoriaceum* and *Buddleia cordata,* respectively. The results of the antinociceptive effects of acetic acid-induced abdominal writhing in mice indicated that oral administration (10 to 200 mg/kg) of *C. subcoriaceum* extract and pectolinarin inhibited the acid-induced writhing in a dose-dependent manner, with an ED_50_ value of 83.2 mg/kg and 28.4 mg/kg, respectively. It is remarkable that pectolinarin exhibits a protective action (76.7%) at the dose 100 mg/kg, similar to the known peripheral analgesic acetylsalicylic acid (70.1%) at the same dose. A higher protective effect (93.1%) was observed for pectolinarin at the dose of 200 mg/kg, compared with morphine sulfate (87.8%) at 1.2 mg/kg. It was therefore established that pectolinarin exhibits potent central analgesic properties, apparently morphinomimetic, associated with its acute anti-inflammatory and peripheral-analgesic activities [15].

The methanol extract of *Cirsium chanroenicum* and the ethyl acetate eluted fraction inhibited cyclooxygenase-2 (COX-2)-mediated prostaglandin E2 (PGE2) and 5-lipoxygenase (5-LOX)-mediated leukotriene production in lipopolysaccharide-treated RAW 264.7 cells and in rat basophilic leukemia (RBL-1) cells treated with A23187calcium ionophore, respectively. In detail, the bio-guided fractionation on silica gel column chromatography of the ethyl acetate fraction led to the isolation of pectolinarin, along with pectolinarigenin. The latter one strongly inhibited COX-2 mediated PGE2 and 5-LOX-mediated LT production at >1 μM, emerging as a dual inhibitor of COX-2/5LOX. However, it did not affect the COX-2 expression or nuclear transcription factor (NF-kB) activation. By experiments in animal models of inflammation/allergy, oral administration of both compounds (20–100 mg/kg) inhibited likewise arachidonic acid-induced mouse ear edema, carrageenan-induced mouse paw edema and passive cutaneous anaphylaxis. It was concluded that pectolinarigenin and pectolinarin possess anti-inflammatory properties and that they may inhibit eicosanoid formation in inflammatory lesions [128].

The evaluation of anti-inflammatory, antipyretic and analgesic effects of *Lawsonia inermis* leaves ethanolic extract, showed that it reduced the rat paw edema significantly at a dose of 1 g/kg, with values of 40.5 ± 4.2 and 55.3 ± 6.1% (*p* < 0.05) at 2 and 4 hours after administration, respectively. However, the analgesic effects did not reach significant levels [254]. In order to identify the responsible agent for anti-inflammatory properties of *L. inermis* leaves, its methanol extract (300 mg/kg b.w) has recently shown an inhibition of paw edema of 3.4 ± 0.2 cm after 3 hours of administration. As one of the isolated flavonoids, pectolinarigenin inhibited the induced inflammatory response to carrageenan by 3.3 ± 0.1 cm, after 3 hours of injection. Pectolinarin instead, resulted in less potent inhibiting carrageenan-induced inflammatory by 3.5 ± 0.1 cm [55]. In summary, all these data suggested a stronger activity of pectolinarigenin than pectolinarin. By wide structure-relationship studies on flavonoids and their anti-inflammatory activity, some preferred structural aspects were determined as follows: (i) the presence of C2=C3 double bond results in a larger volume/surface ratio of the anti-inflammatory effect [255]; (ii) 5-hydroxylation and ring B catechol moiety provides effects for inducing cell differentiation [255]; (iii) methoxylation greatly enhances anti-inflammatory property [256]; and (iv) glycosylation decreases the activity [257], so that the presence of a glycosyl unit in pectolinarin structure supports its higher activity than pectolinarigenin.

More than 400 million people worldwide are suffering from diabetes, with expectations that the number will exceed 640 million by the year 2040 [258]. In recent years, considerable attention has been given to hypoglycaemic agents deriving from plants products, especially on potent antioxidant flavonoids showing positive effects in the management of diabetes mellitus, in improving glucose metabolism and in their action on targets involved in type 2 diabetes mellitus such as α-glycosidase and DPP-4 [242,243,244,245,246,247,248,249,250,251,252,253,254,255,256,257,258,259]. Many reports on animal, cellular and some in vivo studies provide evidence about the beneficial actions of flavonoids to deal with diabetic complications, despite the fact that molecular and cellular mechanisms are not fully elucidated yet. The flavonoids are particularly the ones present in our daily diet, like quercetin, luteolin, baicalein, and acacetin [260].

A study on the inhibition of α-glucosidase and α-amylase involving 21 natural flavonoids, showed that pectolinarin hardly inhibited α-glucosidase and α-amylase at a dose of 5 mg/mL [261]. The α-glucosidase inhibition assay on extracts of *Cirsium japonicum* roots, displayed a considerable activity for the water extract, ranging from 20.1% to 32.6% on α-glucosidase from 10 to 1000 μg/mL and increasing steadily with increasing sample concentrations, whereas the methanol extract had no inhibitory activity at the tested concentrations [262]. Based on these results, a study on the in vivo anti-diabetic activity of a mixture of pectolinarin and pectolinarigenin (62.8% and 36.5%, resp.) aimed to identify the responsible agent for *C. japonicum* effect. The treatment with a dose of 50 mg/kg body weight/day displayed a significant anti-hyperglycemic effect, mainly observed in the case of the flavone mixture. After treatment with pure pectolinarin or pectolinarigenin at the same dose, the increased level of plasma glucose in diabetic rats was observed to decrease by 24.5% and 19.6%, respectively. However, the level in diabetic rats treated with the mixture of pectolinarin and pectolinarigenin (62.8% and 36.5%, resp.) was decreased by 44.7% to be 167.3 ± 13.5 mg/dL. The plasma cholesterol level decreased to 2.5 ± 0.1 and 2.6 ± 0.1 mM in the pectolinarin and pectolinarigenin-treated diabetic rats, while a lower decrease in 29.1% to 2.1 ± 0.1 mM of mixture-treated diabetic rats was observed, in comparison with those of pectolinarin- or pectolinarigenin-treated diabetic rats. Similar results were observed for the plasma triglyceride level, obtaining values of 2 ± 0.1 mM, 1.9 ± 0.1 mM and 1.7 ± 0.1 mM in pectolinarin, pectolinarigenin and the mixture-treated diabetic rats, respectively. These data suggest that pectolinarin and pectolinarigenin improved the severely dysregulated adiponectin expression in diabetic rats, probably leading to a reversal of the altered activities of key metabolic enzymes in liver and ultimately producing an improved glucose homeostasis. On the other hand, the possibility that the typical insulin/insulin receptor signaling pathway was involved in the antidiabetic function of the flavones was excluded [263]. Going on their study, Liao et al. observed that pectolinarin and pectolinarigenin enhanced adipocyte differentiation by increasing the PPARγ transcriptional activity. Additionaly, these flavones promoted both basal and insulin-stimulated glucose uptake in 3T3-L1 adipocytes. A possible explanation was proposed through increased adiponectin and GLUT4 expression and GLUT4 translocation, which was at least partially related to the modulation of insulin signaling [264].

A work regarding the in vitro and in vivo antidiabetic activity of *Kickxia ramosissima* constituents revealed that pectolinarin and pectolinarigenin showed interesting effects. In detail, the two metabolites were more active than the other constituents in the bovine serum albumin (BSA)-glucose test (IC_50_ values of 0.8 and 2.3 mM, respectively), a result which was consistent with the high activity of the ethyl acetate fraction (IC_50_ 88 μg/mL). This inhibition was mainly due to the mode of non-oxidative inhibition as evident in the BSA-methylglyoxal modified albumin (MGO) assay, based on the obtained IC_50_ values (0.19 mM for pectolinarigenin and 0.1 mM for pectolinarin) that were lower than the ones obtained by glucose-BSA test. The α-glucosidase inhibition test showed that pectolinarigenin had the highest activity (IC_50_ = 0.2 mM), similar to acarbose, while pectolinarin exhibited a moderate inhibition of α-glucosidase (48% inhibition at the highest test concentration). On the other hand, in the 15-lipoxygenase inhibition test, pectolinarin displayed the strongest inhibition (IC_50_ = 0.3 mM), in comparison with pectolinarigenin and the other *Kickxia ramosissima* constituents [202]. All these results can also be considered as support for the potent antidiabetic activity observed for *Linaria reflexa* methanolic extract, which contains in high amounts several pectolinarin and pectolinarigenin derivatives. Specifically, the evaluation reported by Cheriet et al. on the in vivo antidiabetic activity of *Linaria reflexa* extract showed a remarkable effect at the dose 300 mg/kg, allowing the most marked reduction of the glycemia of alloxan diabetic rats (−72.1%), when compared with the effects of the drug glybenclamide (−63.3%) [74]. Oral administration of acacetin (3 and 31.6 mg/kg), which is structurally similar to pectolinarigenin (lacking of the OMe-6 group), has been reported to cause a significant decrease in blood glucose levels in both healthy mice and those suffering from hyperglycemia when compared with vehicle-treated groups [265]. Previous studies suggested that hydroxylation in position 7 provided compounds, which behaved as PPAR agonists in vitro [266,267], explaining the remarkable effect observed for pectolinarigenin.

### 3.3. Cytotoxic and Antitumor Activities

Cancer is nowadays one of the most serious life-threating diseases, affecting people of all ages, and is considered one of the leading causes of mortality and morbidity worldwide [268]. Plant metabolites are characterized by peculiar molecular structures, promising to be a peculiar source of anticancer agents. In detail, a series of studies have also evaluated the antitumor activities of pectolinarin and pectolinarigenin. In vitro investigation on the antiproliferative activity of several flavonoids related to pectolinarigenin isolated from *Linaria reflexa*, showed for pectolinarin a high cytotoxic effect on lung carcinoma (COR-L23), colorectal adenocarcinoma (Caco-2) and amelanotic melanoma (C32) cell lines (IC_50_ 5.1, 6.2 and 7.2 μM, respectively), while pectolinarigenin displayed the highest effect on COR-L23 and C32 cells (IC_50_ 4.1 and 7 mM, respectively) [72]. These results encouraged the establishment of a structure activity relationship of pectolinarigenin involving some synthetic derivatives. The analogue showing a dimethylamino-propoxy group in *O*-7 position was identified as the most active, with IC_50_ values of 7.2 and 7.4 μM against COR-L23 and A549 cell lines, respectively. Conversely, none of the other synthetic derivatives exerted a relevant effect. Natural pectolinarigenin exhibited instead a potent activity against malignant melanoma (A375) and Caucasian lung carcinoma (A549) (IC_50_ 8.2 ± 1.3 and 5.6 ± 0.9 μM, respectively) [269].

Pectolinarigenin has also shown a potent activity against breast cancer, which is the most commonly diagnosed cancer type in women worldwide [270]. Fesearch by Lu et al. [271] dealt with the antiproliferative activity of pectolinarigenin isolated from *Cirsium japonicum*, against breast cancer cells, inhibiting the MCF-7 colony formation almost completely inhibited at 50 μM. The same study demonstrated that pectolinarigenin induced apoptosis and downregulation of Bcl2 expression, with a complete inhibition at the dose 25 mM. Pectolinarigenin was also observed to inhibit tumor cell self-renewal. The treatment with pectolinarigenin at a 25 μM concentration caused a significant inhibition of colony formation (61.2%, *p* < 0.001) and tumor sphere formation (59.5%, *p* < 0.01) in MCF-7, besides changes in breast cancer stem cell markers, and reduction of the chemoresistance of the cells to doxorubicin. In addition, mRNA expression of chemoresistance genes (ATP binding cassette subfamily G member 2, ABCG2 and ATP binding cassette subfamily B member 1, MDR1) was suppressed. A treatment with 10 μM pectolinarigenin inhibited the MDR1 and ABCG2 efficinecy of about 59.3 (*p* < 0.01) and 46.5% (*p* < 0.01), respectively and, moreover, tumor mass in nude mice xenograft model was reduced. By these data, pectolinarigenin emerges as a promising agent in treatment of patients with breast cancer [129]. Recent epidemiological studies suggest also that the consumption of healthy diets, with fruits and vegetables rich in flavonoids, and control of body weight, lead positively to reduce the incidence of breast cancer [272].

Beside the interesting and promising activity against breast cancer, which requires further studies, pectolinarigenin was investigated in the inhibition effect on nasopharyngeal carcinoma cell line (NPC C666-1), causing a rare type of head and neck cancer. It showed cytotoxic effects and induced apoptosis of the C666-1 cells through mitochondria-related apoptosis and ROS-induced apoptotic pathways. The in vivo experiment on the subcutaneous xenograft mice model indicated that a pectolinarigenin administration could decrease the tumor growth of NPC and no severe toxicity was observed [273]. With the aim to explore the potential molecular mechanism of pectolinarigenin, its effect was investigated against signal transducer and activator of transcription 3 (STAT3), which is an attractive target for cancer therapy, being an important transcription factor involved in proliferation, survival, apoptosis, angiogenesis, and metastasis [274]. Therefore, an investigation on osteosarcoma growth and metastasis via SHP-1-mediated STAT3 signaling inhibition showed that pectolinarigenin inhibited constitutive and interleukin-6-induced STAT3 signaling, decreased the accumulation of STAT3 in the nucleus and blocked STAT3 DNA-binding activity in osteosarcoma cells, as well as disturbing the DNMT1/HDAC1/STAT3 complex formation in a SHP-1 promoter site, therefore, releasing the transcription repression of SHP-1. These findings demonstrate that the pectolinarigenin action mainly dependes on SHP-1-mediated STAT3 signaling suppression and provides solid evidence for its anti-osteosarcoma action, supporting its potential as an anticancer agent [274].

Regarding the anti-tumor immune therapy (BRMs), a study on a mixture of pectolinarin and pectolinarigenin isolated from *Cirsium japonicum* was evaluated. A significant difference of immune response between the mixture-treated tumor-bearing mice and negative control was observed. Pectolinarin, pectolinarigenin and their mixture inhibited the growth of the implanted tumors and promoted complement hemolytic (CH50) activity in the tumor-bearing mice, improving also the spleen cell transformation and NK cell activity. The mixture of the two metabolites gave a better tumor-inhibiting and immune-promoting effect than each single flavone, showing the function of protecting immune cells and reversing the pathway of apoptosis in immune cells induced by tumor cells. The most remarkable effect against mouse liver cancer (H22) and mouse sarcoma (S180) was observed at each dose 50 mg/kg of the mixture, pectolinarin and pectolinarigenin, obtaining tumor inhibition values of 53.2%, 41.7%, 37.4% and 50.5%, 39.6%, 35.9%, respectively [275]. Furthermore, these data were enriched with a high similarity in results by Liu et al. [7]. Pectolinarigenin, isolated from *Clerodedrum indicum* roots and stems extract, showed a good cytotoxicity with an LC_50_ of 4.3 mg/mL [215]. In 2017, Zhou et al. reported how pectolinarigenin suppresses pancreatic cancer cells (Patu 8988 and BxPC-3) growth by inhibiting STAT3 signaling. Meanwhile, according to the results from colony formation and wound healing assays, pectolinarigenin was able to inhibit cell viability and cell migration, as well induced apoptosis and decreased phosphorylation of STAT3, modulating its signaling module, and thereby inducing cytotoxicity in pancreatic cancer cells [276].

Very recently, Wu et al. [277] investigated the potential molecular mechanisms of pectolinarigenin on hepatocellular carcinoma (HCC) cells. The results indicated that the treatment with pectolinarigenin (10 µM) significantly inhibited cell proliferation and migratory and invasive abilities in a concentration- and time-dependent manner. In detail, the natural product induced apoptosis and caused G2/M phase arrest of HCC via PI3K/AKT/mTOR/ERK signaling pathway. Furthermore, it significantly suppressed hepatocellular carcinoma tumor growth in vivo.

The encouraged data on the tumor inhibition By pectolinarigenin motivated the investigation of its mechanism of action on different cancer types. A recent work by Lee et al. [278] deals with the mechanism of pectolinarigenin-induced cell death caused by autophagy and apoptosis in AGS and MKN28 human gastric cancer cells. A novel mechanism of pectolinarigenin was elucidated to induce G2/M arrest, apoptosis, and autophagy in vitro. It was clarified that the PI3K/AKT/mTOR pathway plays a vital role in pectolinarigenin-induced cell death in human gastric cancer cell, which makes this flavone a future probable anticancer therapeutic agent [279]. A further evaluation of pectolinarigenin as able to inhibit human non-small cell lung cancer cell proliferation, metastasis and epithelial–mesenchymal transition, as well as to promote apoptosis, has also been reported through the PTEN/PI3K/AKT signaling pathway [280]. The inactivation of the phosphatidylinositol 3 kinase/protein kinase B (PI3K/AKT) pathway was recently observed also for pectolinarin during its study on the capacity to suppress cell proliferation and inflammatory response, and induce apoptosis in rheumatoid arthritis fibroblast-like synoviocytes [281].

With the aim of searching for new drugs able to inhibit tumor metastasis, especially in the case of colorectal carcinoma (CRC), an in vitro and in vivo study was carried out on pectolinarigenin to determine its underlying mechanism of action. The treatment with pectolinarigenin could inhibit cancer cell growth and induce apoptosis, block cell migration and invasion, impairing CRC cell migration and invasion by downregulating the expression of MMP9 and phosphorylated-Stat3Tyr705. The observed results suggest that pectolinarigenin is a potential therapeutic agent for inhibiting colorectal carcinoma growth and metastasis in a concentration- and time dependent manner [282].

Notably, many reports have provided evidence about the role of C2=C3 double bond [283], 6-OH and 5,7-diOH groups in improving the biological effects [284,285], while glycosylation decreases the activity in comparison with aglycones [286]. Pectolinarigenin is characterized by containing most of these structural moieties, which can explain its stronger effect than the corresponding glycosylated pectolinarin.

### 3.4. Other Biological Activities

#### 3.4.1. Antiviral

Currently, a series of drugs for diseases caused by herpes viruses, retroviruses, orthomyxoviruses, hepatitis B virus, and hepatitis C virus (HCV) are commercially available [287]. However, the development of novel antiviral agents is necessary due to the high recrudescence of these infections for which there are no specific treatment and to the constant apparition of new resistant viral strains. Few reports have been published on flavonoids activity against RNA viruses, such as influenza A virus, human immunodeficiency virus (HIV), rotavirus, poliovirus, coxsackievirus, dengue fever virus, and Japanese encephalitis virus [288,289,290,291,292,293]. Moreover, the structure–activity relationship is not yet cleary demonstrated [294]. A study on the ethanolic extract obtained from *Distictella elongata* and its isolated constituents, against human herpesvirus type 1 (HSV-1), murine encephalomyocarditis virus (EMCV), vaccinia virus western reserve (VACV-WR) and dengue virus 2 (DENV-2), showed that the most remarkable effect was observed for a mixture of pectolinarin with acacetin-7-*O*-rutinoside against DENV-2 was (EC_50_ 11.1 ± 1.6, CC_50_ > 500 μg/ml), whereas pure pectolinarin was less active against DENV-2 (EC_50_ 86.4 ± 3.8, CC_50_ 402.6 ± 9.8 μg/ml), VACV-WR (EC_50_ 207.1 ± 9.6, CC_50_ 449.0 ± 13.0 μg/mL) and HSV-1 (EC_50_ 314.5 ± 22.7, CC_50_ 449.0 ±13.0 μg/mL) [295].

The flavonoids’ activity against Corona-viruses has been directly associated to the inhibition of 3C-like protease (3CLpro). In a study on some flavonoids, pectolinarin has shown to effectively block the enzymatic activity of SARS-CoV 3CLpro. Specifically, its interaction was proved using a tryptophan-based fluorescence method, observing a severely reduced fluorescence intensity with an IC_50_ value of 37.8 μM. An induced-fit docking analysis showed also that the hydrogen bond formed between the 7-hydroxyl group of glycosylated flavonoids and the backbone of Ile188 (a rare variant of the human A3C enzyme) was abolished due to the presence of the sugar moiety. All the obtained results suggest that the presence of hydrophobic aromatic rings and hydrophilic hydroxyl groups, together with the presence of carbohydrate groups, severely influences the binding affinity of the chromen-4-one moiety, which is the case of pectolinarin [296].

In most studies, the C2=C3 double bond has been reported as a basic favorable structural feature for antiviral activity; moreover, glycosylated flavonoids exert greater antiviral effects than the aglycone ones [297]. The positive role of 5,7-dihydroxyl derivatives has been observed [293], while methoxylation decreased the activity [298], supporting why acacetin-7-*O*-rutinoside was more active than pectolinarin.

#### 3.4.2. Antiparasitic

A broad set of parasites are using humans as hosts to evolve, usually without killing their host, at least not immediately. Most parasites are regrettable or able to weaken our health, but some parasitic infections (i.e., malaria and trypanosomiasis) can also cause the death of patients when not treated with appropriate therapeutics [299]. Recently, several types of flavonoids have been identified as antiparasitic agents of plant extracts [300,301,302], although a comprehensive study on their structure–activity relationships (SARs) has not been carried out so far.

The anti-leishmanial effects of some compounds from the aerial parts of *Baccharis uncinella* has been investigated. In particular, pectolinarigenin showed a remarkable effect against *Leishmania* (V.) *braziliensis* (IC_50_ 110 ± 3 μg/μL) and exhibited the highest inhibition of the intracellular forms from both *L.* (L.) *amazonensis* and *L.* (V.) *braziliensis* (IC_50_ of 8.0 ± 0.5 and 60.0 ± 0.01 ng/μL, respectively) [117].

An in vitro and in vivo evaluation on the inhibitory effects of some flavonoids on P-glycoprotein (P-gp) showed for pectolinarigenin (100 μM) a moderate activity with permeability coefficient values of 2.1 ± 0.5, 10.3 ± 1.3 (×10^−6^ cm/s), causing an inhibition of 44.8%. This effect was related to the presence of hydroxyl or methoxyl groups at the 6- and 7-positions in the aromatic B-ring (Figure 1) [303].

The effects and mechanisms of the flavonoids present in *Linaria vulgaris* and previously reported to inhibit in vitro lipid accumulation, were investigated on hyperlipidemia and hepatic steatosis induced by a Western type diet. The plant extract rich in flavonoids (especially pectolinarin, isolinariin A and B) exhibited a protective effect against hyperlipidemia and hepatic steatosis induced by a Western-type diet. Kuang et al. suggested that the observed activity was related to the presence of flavonoids showing a methoxyl group in the A-ring [304], previously reported to exhibit lipid-lowering effects [305] and identified as pectolinarin, isolinariin A and B. The mechanism might be due to the suppression of mature nuclear form of sterol regulatory element-binding protein (n-SREBP) expressions and modulation of expression of its target gene caused by flavonoids treatment, thereby resulting in a relatively normal lipid level in serum and liver [304].

## 4. Conclusions

In recent decades, traditional herbal medicines have become a promising direction for the development of new therapeutic agents to treat a range of serious diseases, including inflammation, diabetic disorder and cancer. In the wide family of flavonoids, a number of reports has been published on the isolation of pectolinarin and pectolinarigenin, and on their biological evaluation. They have shown antimicrobial activity and antioxidant property, the latter one related to impressive anti-inflammatory, antidiabetic and antitumor activities, which made these metabolites a new target for a full understanding of the mechanism of action. While pectolinarin resulted in being more efficient as antidiabetic, its aglycone pectolinarigenin has shown a potent antitumor activity escpecially against breast cancer. A series of structure–activity relationship studies have been carried out to identify the molecular peculiarities responsible for the bioactivities, which are useful for further lead optimization. Moreover, recent epidemiological studies have suggested that the consumption of diets rich in fruits and vegetables containing flavonoids, contributes positively to reduce the incidence of some types of tumors.

## Figures and Tables

**Table 1:** Isolation of pectolinarin from the indicated plants, classified according to family, genus and species, and place of collection.

Genus	Species	Collection Place	Reference
**Family:** Adoxaceae			
*Viburnum*	*V. cotinifolium*	Kashmir/India	[13]
	*V. mullaha*	Indian Himalayan region	[14]
**Family:** Asteraceae			
*Cirsium*	*C. subcoriaceum*	Pahuatlan/Mexico	[15]
	*C. japonicum*	Daejeon/S. Korea, Oberndorf/Austria, Chengdu/China, Henan/China	[7,16,17,18,19,20]
		Oberndorf/Austria	
		Bialystok/Poland	
		Daejeon/S. Korea	
	*C. setosum*	Daejeon/S. Korea	[18]
	*C. rivulare*	Daejeon, Wonju, Pyongchang-gun, Gangwondo, Jeongseon-gun, Yanggu/S. Korea	[21]
	*C. lineare*	Daejeon/S. Korea	[19]
	*C. nipponicum*	Daejeon, Sancheong/S. Korea	[19,22]
	*C. setidens*	Jeongseon-gun, Jeju Island/S. Korea	[19,23,24,25,26]
		Laramie/USA	
		Laramie/USA, Vitebsk/Belarus	
	*C. pendulum*	Nemuro, Hatimandake, Memanbetsu, Onsen Kyushu, Hokkaido/Japan	[19]
	*C. chanroenicum*		[19,26,27]
	*C. rhinoceros*		[28,29]
	*C. coloradense*	Wyoming/USA, Japan	[30]
	*C. arisanense*	Mount Akaishi, Mount Senmai, Shizuoka, Takanomori, Nekura Valley/Japan, Vitebsk/Belarus,	
	*C. tioganum*	Mount Shirouma/Japan	
	*C. oleraceum*	la Dotze/Switzerland	[30,31,32]
		Hsien/Taiwan	
	*C. microspicatum*	Nemuro, Mount Shirouma /Japan, Mount Ali, Chiayi Hsien/Taiwan	[16]
	*C. babanum*	Ku Kuan, Taichung Haien/Taiwan	
	*C. kagamontanum*	Mount Shirouma, Mount Hakusan ad pedem/Japan	
	*C. inundatum*	Vancouver/Canada	
	*C. dipsacolepis*	Seongnam/Korea	
	*C. brevicaule*		
	*C. yezoense*		
	*C. kamtschaticum*		
	*C. pectinellum*		
	*C. bitchuense*		[33,34]
	*C. senjonse*		[17,31,35,36,37]
	*C. spicatum*		
	*C. yezonese*		
	*C. vallis-demonii*		
	*C. gratiosum*		
	*C. indundatum*		
	*C. otayae*		[35]
	*C. purpuratum*		
	*C. spinosissimum*		[38]
	*C. spinosum*		[34]
	*C. ferum*		[39]
	*C. kawakamii*		[16,40,41]
	*C. wallichii*		[41]
	*C. yoshizawae*		[35]
	*C. matsumurae*		[34,40]
	*C. brevistylum*		[42]
	*C. chlorolepis*		[43]
*Duranta*	*D. plumieri*	Rajshahi/Bangladesh	[44]
*Hemistepta*	*H. lyrata*	Kangwon/S. Korea	[45]
*Picnomon*	*P. acarna*	Mount Hortiatis/Greece	[46]
**Family:** Bignoniaceae			
*Distictella*	*D. elongata*	Minas Gerais State/Brazil	[47]
*Markhamia*	*M. lutea*	Benguluru/India	[48]
**Family:** Buddlejaceae			
*Buddleja*	*B. officinalis*	Anhui/China	[49]
Family: Ericaceae			
*Rhododendron*	*R. arboreum*	Aligarh/India	[50]
**Family:** Gesneriaceae			
*Corallodiscus*	*C. flabellate*	Kunming/China	[51]
*Aeschynanthus*	*A. moningeriae*	Jinhua/China	[52]
**Family:** Lamiaceae			
*Leucosceptrum*	*L. canum*	Tibet/China	[53]
*Teucrium*	*T. hyrcanicum*	Sicily/Italy	[54]
**Family:** Lythraceae			
*Lawsonia*	*L. inermis*	Thanjavur/India	[55]
**Family:** Moraceae			
*Clerodedrum*	*C. phlomides*	Tamil Nadu/India	[56]
**Family:** Orchidaceae			
*Oncidium*	*O. baueri*	Londrina/Brazil	[57,58]
**Family:** Orobanchaceae			
*Melampyrum*	*M. roseum*	Suwon/S. Korea	[59]
**Family:** Plantaginaceae			
*Scoparia*	*S. dulcis*	Nanning/China	[60]
**Family:** Poaceae			
*Oryza*	*O. sativa*	a)	[61]
**Family:** Ranunculaceae			
*Trollius*	*T. ledebourii*	Hebei/China	[62]
**Family:** Rosaceae			
*Kerria*	*K. japonica* var.	Chongqing/China	[63]
*Crataegus*	*C. laevigata*	Bremen/Germany	[64]
**Family:** Santalaceae			
*Thesium*	*T. chinense*	Anhui/China	[65]
**Family:** Scrophulariaceae			
*Linaria*	*L. vulgaris*	Sofia/Bulgaria, Tachkent/Uzbekistan	[2,66,67,68]
	*L. japonica*	Heilongjiang/China	[69,70,71]
	*L. reflexa*	Tottori Prefecture/Japan	[72,73,74,75]
	*L. vulgariformis*	Constantine/Algeria, Calabria/Italy	[66]
	*L. popovii*	Tachkent/Uzbekistan	
	*L. kurdica*		
	*L. sessili*		[76]
	*L. kokanica*	Pamir/Tajikistan	
	*L. haelava*		[77]
	*L. simplex*	Mansoura/Egypt	[67]
	*L. genistifolia*	Sofia/Bulgaria	
	*L. dalmatica*		
	*L. scariosa*	Msila/Algeria	[78]
*Kickxia*	*K. elatine*	Dustlik/Uzbekistan	[79]
	*K. heterophylla*	Mansoura/Egypt	[80]
	*K. ramosissima*	Ankara/Turkey	[81,82]
	*K. abhaica*	Baljurashi/Saudi Arabia	[83]
		Appennines hills/Italy	
	*K. spuria*	Saudi Arabia	[84]
	*K. aegyptiaca*		[85]
**Family:** Verbenaceae			
*Morus*	*M. alba* L.	Hongseong/Korea	[86]
*Lantana L. camara*		Taichung/Taiwan, Palampur/India, Karachi/Pakistan, Ceará state/Brazil, Manado/Indonesia, Okinawa/Japan	[87,88,89,90,91,92]
*Lippia L. rubella*		Minas Gerais/Brazil	[93]
Family: Winteraceae			
*Tasmannia*	*T. lanceolata*	Go Wild Harvest/Australia	[94]

^a^ Not found.

**Table 2:** Isolation of pectolinarigenin from the indicated plants, classified according to family, genus and species, and place of collection.

Genus	Species	Collection Place	Reference
**Family:** Apiaceae			
*Coriandrum*	*C. sativum*	Faisalabad/Pakistan	[99]
**Family:** Aspleniaceae			
*Asplenium*	*A. glaucophyllum*	West Malysia	[100]
	*A. normale*	West Malaysia	[101]
**Family:** Asteraceae			
*Achillea*	*A. collina*	wet lowlandmeadows/UK	[102]
	*A. asplenifolia*		
*Ajania*	*A. potaninii*	Gansu/China	[103]
*Ambrosia*	*A. camphorate*	Baja California/Mexico	[104]
*Arnica*	*A. angustifolia*	northwestCanada and Alaska	[105]
	*A. Montana*	California/USA	[106]
	*A. chamissonis*	Graines Voltz/France	[107]
	*A. montana*	Šumava Mounts/Czech	[108]
*Artemisia*	*A. mongolica*	Gansu/China	[109]
	*A. judaica*	St. Catherine, Sinai/Egypt	[110]
	*A. monosperma*	Cairo/Egypt	
	*A. herba-alba*	Mount Moses/Egypt	
	*A. xerophytica*	South Gobi Aimak/Mongolia	[111]
	*A. glabella*	Karaganda/Kazakhstan	[112]
	*A. vestita*	Lhasa/Tibet	[113]
*Baccharis*	*B. trinervis*	Costa Rica	[114]
	*B. decussata*	Venezuela	[115]
	*B. concave*		[116]
	*B. uncinella*	Campos do Jordão/Brazil	[117]
	*B. conferta*	Veracruz/Mexico	[118]
*Centaurea*	*C. alexandrina*	Alexandria/Egypt	[119]
	*C. aspera*	Ribera Baixa/Spain	[120]
	*C. cariensis*		[121]
	*C. collina*	Valencia/Spain	[122]
	*C. sadleriana*	Jakabszállás/Hungary	[123]
	*C. moesiaca*	Malashevska planina/Bulgaria	[124]
	*C. behen*	Iran	[125]
*Chromolaena*	*C. odorata*	Chonburi/Thailand	[126]
*Chrysanthemum*	*C. pacificum*	Tsukuba/Japan	[27]
	*C. shiwogiku*	Muroto-misaki/Japan	
	*C. kinokuniense*	Tsukuba/Japan	
	*C. rupestre*	Mount Mikuni/Japan	
*Cirsium*	*C. setidens*	Jeongseon-gun, Halla of jejudo, Daejeon, Kangwon, Yanggu/S. Korea; Guerrero/Mexico;	[19,24,25,26,27,28,127]
	*C. chanroenicum*	Daejeon, Ulsan, Sancheong/S. Korea	[26,128]
	*C. japonicum*	Jiang Xi/China	[129]
	*C. arvense*	Musa Khel Bannu/Pakistan	[130]
	*C. nipponicum*	Suwon/S. Korea	[131]
	*C. rhinoceros*		[132]
*Dichrocephala*	*D. integrifolia*	Shanghai/China	[133]
*Dugaldia*	*D. pinetorum*	Nuevo Lebn/Mexico	[134]
*Eriocephalus*	*E. giessii*	Aus-Koppies/Namibia	[135]
*Eupatorium*	*E. cannabinum*	Gronigen/Netherlands	[136]
	*E. odoratum*	Kuala Pilah/Malaysia	[137,138]
	*E. semiserratum*	Arkansas/USA	[139]
*Fragrant*	*F. Eupatorium*	Guangxi/China	[140]
*Grindelia*	*G. glutinosa*	Poconchile, Valle deLiuta, Tarapaca/Chile	[141]
*Gutierrezia*	*G. mandonii*	Salta/Argentina	[142]
*Helenium*	*H. integrifolium*		[143]
*Heterotheca*	*H. latifolia*	San Luis/Argentina	[144]
*Hemistepta*	*H. lyrata*	Kangwon/S. Korea	[45]
*Hymenoxys*	*H. jamesii*	Coconino/USA	[145]
*Iva*	*I. nevadensis*	Tonopah/USA	[146]
	*I. frutescens*	Franklin/USA	[147]
*Jungia*	*J. polita*	San Martin/Argentina	[148]
*Olearia*	*O. paniculata*	Dunedin/New Zealand	[149]
*Onopordon*	*O. corymbosum*	Barracas, Castellon/Spain	[150]
	*O. nervosum*	a)	[151]
*Santolina*	*S. chamaecyparissus*	Lyon/France	[152]
	*S. pinnata*	Pisa/Italy	[153]
*Saussurea*	*S. elegans*	Murghab/Tajikistan	[154]
*Schkuhria*	*S. pinnata*	Cordoba/Argentina	[155]
*Seriphidium*	*S. santolium*	Xinjiang Uigour/China	[156]
*Stevia*	*S. laxiflora*	Cuernavaca, Morelos/Mexico	[157]
*Vernonia*	*V. cinerea*	Pahang/Malaysia	[158]
**Family:** Betulaceae			
*Alnus*	*A. glutinosa*	Darmstadt/Germany	[159]
	*A. japonica*		[160]
*Betula*	*B. ermanii*		[159]
	*B. verrucosa*	a)	[161]
	*B. pubescens*	Biebrza/Poland	[162]
	*B. pendula*		
**Family:** Bignoniaceae			
*Millingtonia*	*M. hortensis*	Khon Kaen/Thailand	[163]
**Family:** Blechnaceae			
*Brainea*	*B. insignis*	Yunnan/China	[164]
**Family:** Boraginaceae			
Eriodictyon	*E. tomentosum*	Placer Co./USA	[165]
**Family:** Fabaceae			
*Adesmia*	*A. grandiflora*	a)	[166]
	*A. trijuga*		
	*A. horrida*		
	*A. retrofracta*		
*Ononis*	*O. fruticosa*	Los Castanõs/Spain	[167]
	*O. natrix*		
	*O. rotundifolia*	a)	[168]
*Trifolium*	*T. pratense*	Trout Lake/USA	[169]
**Family:** Lamiaceae			
*Leucosceptrum*	*L. canum*	a)	[170]
*Mentha*	*M. pulegium*	Petite Kabylie/Algeria	[171]
	*M. suaveolens*		
*Ocimum*	*O. americanum*	RoyalBotanic Gardens, Kew/England	[172]
*Otostegia*	*O. fruticosa*	St. Catherine/Egypt	[173]
*Salvia*	*S. trilobu*	Marmara island/Turkey	[174]
	*S. hypoleuca*	Elbruz moun/Russia	[175]
	*S. pedicellata*	a)	[176]
	*S. yosgadensis*	Sultanhani/Turkey	[177]
	*S. plebeia*	a)	[178]
	*S. pilifera*	Berit Mount/Turkey	[179]
	*S. tomentosa*	Sofia/Bulgaria	[180]
	*S. argentea*		
*Scutellaria*	*S. polyodon*	a)	[181]
	*S. przewalskii*	Susamyr/Kyrgyzstan	[182]
*Sideritis*	*S. gomerae*	Canary islands/Spain	[183]
*Teucrium*	*T. chamaedrys*	Eskisehir/Turkey	[184]
*Thymus*	*T. longicaulis*	Sar planina/Macedonia	[185]
	*T. glabrescens*	Skopje/Macedonia	
**Family:** Lythraceae			
*Lawsonia*	*L. inermis*	Thanjavur/India	[153]
**Family:** Nothofagaceae			
*Nothofagus*	*N. dombeyi*	Altos de Lircay/Chile	[186]
**Family:** Orobanchaceae			
*Striga*	*S. passargei*	a)	[187]
	*S. aspera*	a)	[188]
**Family:** Padaliacea			
*Sesamum*	*S. indicum*	Gambang/Malaysia	[189]
**Family:** Plantaginaceae			
*Digitalis*	*D. trojana*	Kizilcahamam, DemirkOy/Turkey	[190]
	*D. orientalis*		
	*D. lanata*	a)	[191]
*Hebe*	*H. cupressoides*	Dunedin/New Zealand	[192]
*Veronica*	*V. chamaedrys*	Rila Mount/Bulgaria	[193]
*Veronicastrum*	*V. latifolium*	Yongkang/China	[194]
**Family:** Portulacaceae			
*Portulaca*	*P. oleracea*	Tianjin/China	[195]
**Family:** Ranunculaceae			
*Trollius*	*T. chinensis*	Hebei/China	[196]
**Family:** Rosaceae			
*Rosa*	*R. damascena*	Plovdiv/Bulgaria	[197]
	*R. rugosa*	Botanischer Garten der TU Darmstadt/Germany	[198]
**Family:** Scrophulariaceae			
*Buddleia*	*B. macrostachya*	Sibsagar/India	[199]
*Kickxia*	*K. ramosissima*	Takht-e-Nusrati/Pakistan	[200,201,202]
*Limnophila*	*L. aromatica*	Ho Chi Minh/Vietnam	[203]
*Linaria*	*L. vulgaris*	Ukrania; China	[68,204]
	*L. reflexa*	Constantine/Algeria	[73]
	*L. kurdica*	Ukrania	[204]
	*L. scariosa*	Msila/Algeria	[78]
**Family:** Verbenaceae			
*Clerodendrum*	*C. siphonenthus*	Calcutta, Kalyani/India	[205,206]
	*C. phlomidis*	Pondicherry, Alanthurai/India	[205,206,207,208,209,210]
	*C. serratum*	Bhilai/India	[211]
	*C. inerme*	Pondicherry/India	[212,213]
	*C. neriifolium*	a)	[214]
	*C. indicum*	a)Khao Kho/Thailand	[215,216]
*Duranta*	*D. repens*	a)	[217]
	*D. plumieri*	a)	[44,218]
*Lantana*	*L. camara*	Taichung/Taiwan, Palampur/India, Karachi/Pakistan, Ceará state/Brazil, Manado/Indonesia, Okinawa/Japan	[87,88,89,90,91,92]
*Lippia*	*L. citriodora*	Athens/Greece	[219]
**Family:** Zosteraceae			
*Phyllospadix*	*P. japonica*	Omaezaki/Japan	[220]

^a)^ not found.
